# Supplementary material for: Distinct CD1d docking strategies exhibited by diverse Type II NKT cell receptors
Source: Nat Commun. 2019 Nov 20;10:5242. doi: 10.1038/s41467-019-12941-9 (PMC6868179; doi:10.1038/s41467-019-12941-9)
Supplement: Supplementary file 1 — Supplementary Information [file 41467_2019_12941_MOESM1_ESM.pdf]

Supplementary information

Distinct CD1d docking strategies exhibited by diverse Type II NKT cell receptors.

Catarina Almeida et al.

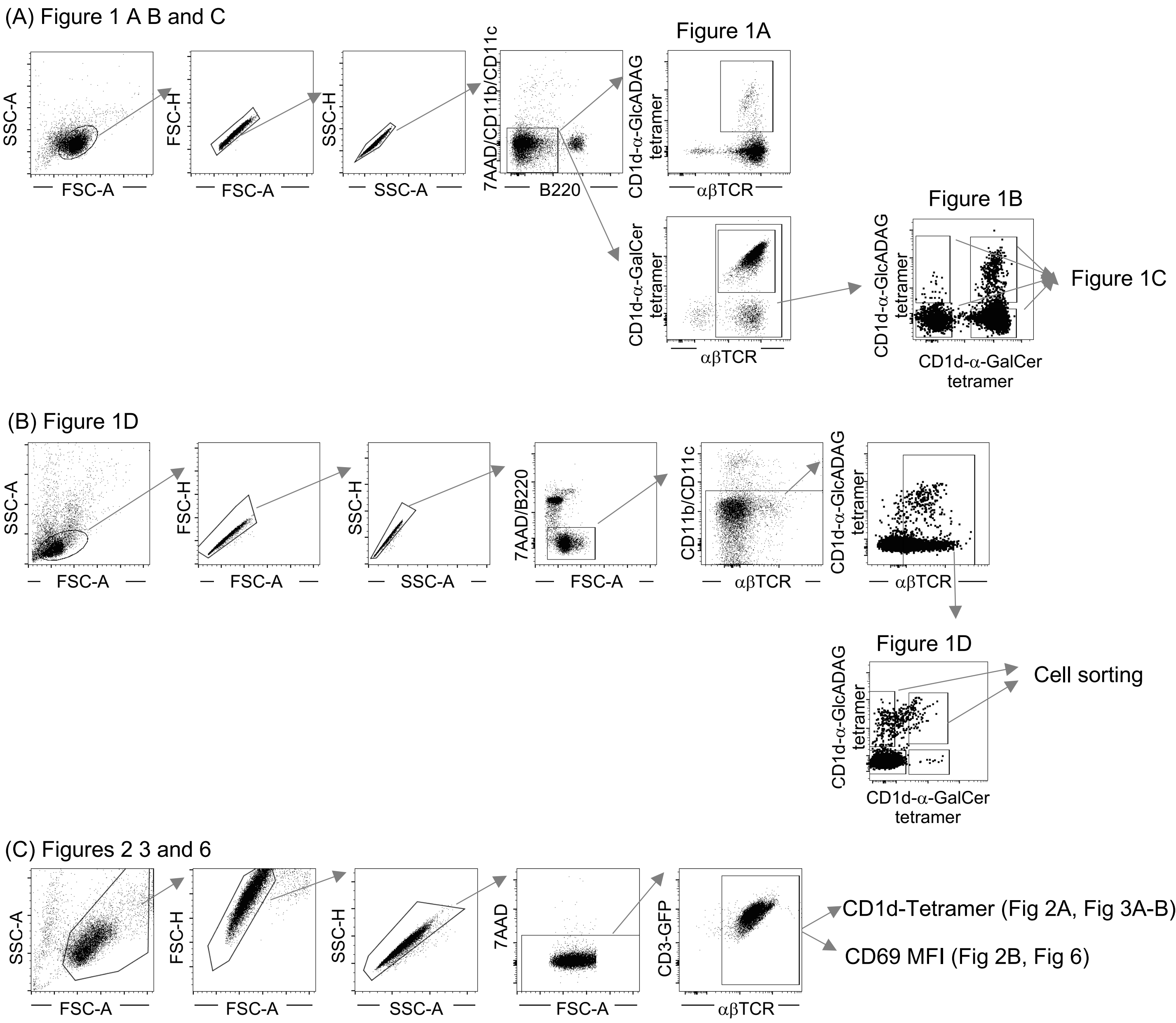

Supplementary Figure 1. Gating strategies used for flow cytometry analysis and cell sorting. (**A**) Gating strategy to identify (7AAD$^-$ B220$^-$ CD11c$^-$ CD11b$^-$) CD1d-α-GlcADAG and CD1d-α-GalCer tetramer+ thymocytes from CD24-depleted BALB/c mice in Figures 1A-C. (**B**) Gating strategy to identify (7AAD$^-$ B220$^-$ CD11c$^-$ CD11b$^-$ αβTCR$^{int/hi}$) CD1d-α-GlcADAG and CD1d-α-GalCer tetramer+ thymocytes from samples that underwent CD1d-α-GlcADAG tetramer-associated magnetic enrichment in Figure 1D and used for single cell paired TCR sequencing analysis in table 1. (**C**) Gating strategy to analyse TCR transduced Bw58 (7AAD$^-$ CD3$^+$αβTCR$^+$) cell lines. The same strategy was used to determine CD1d-tetramer binding or CD69 MFIs for figures 2A, 2B, 3 and 6

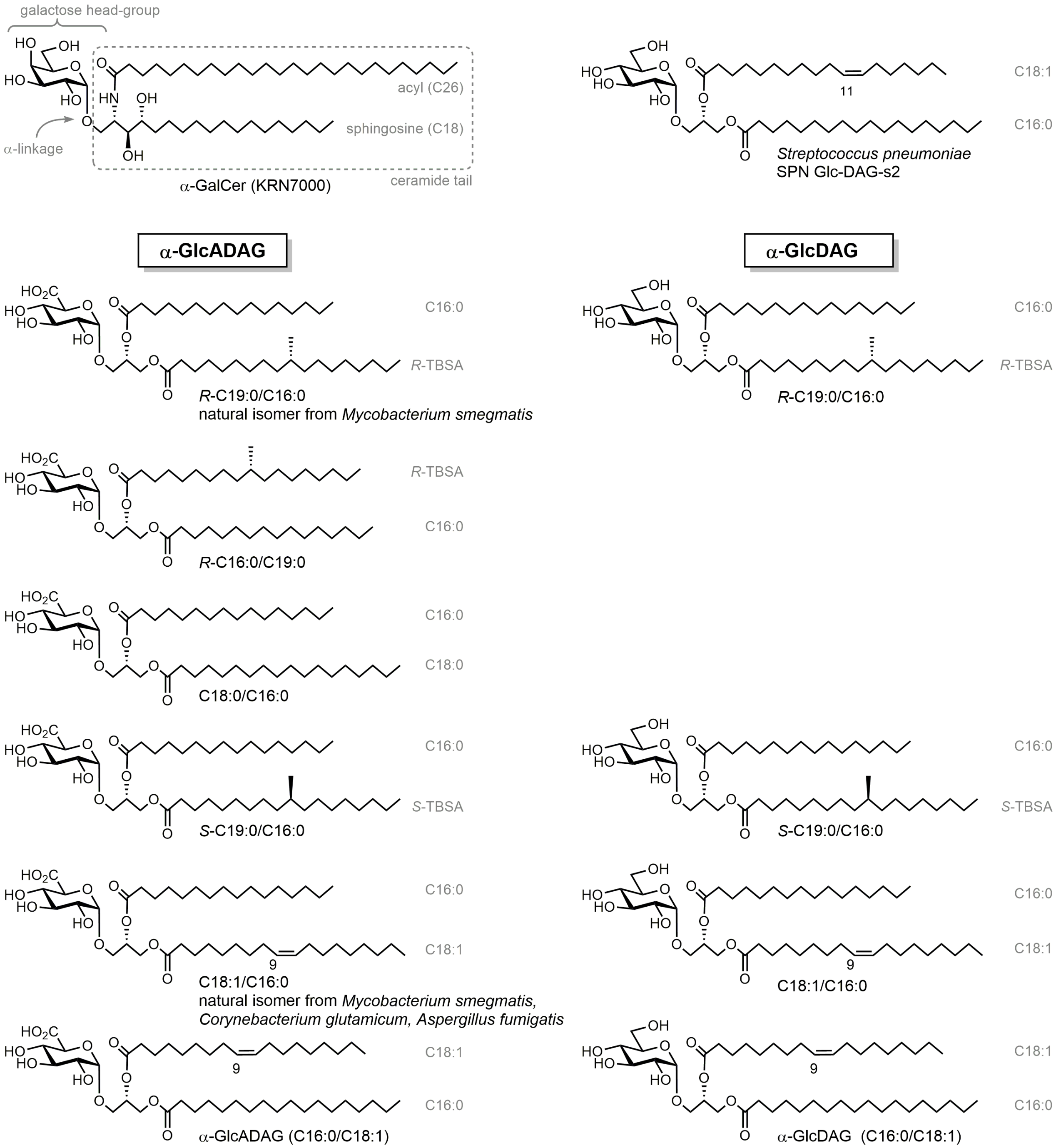

Supplementary Figure 2. Structures of α-GalCer, α-GlcADAG or α-GlcADAG variants.

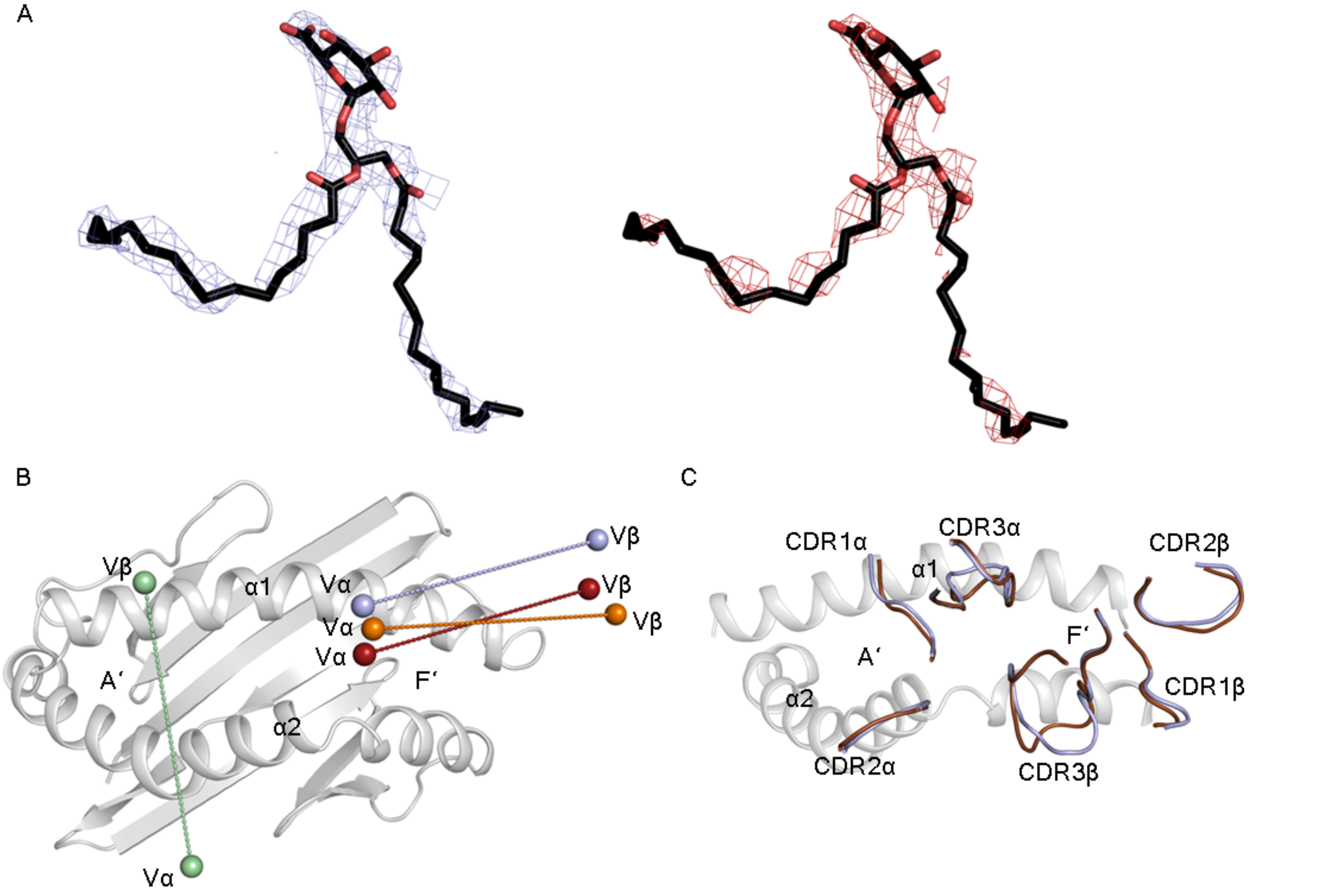

Supplementary Figure 3. (**A**) 2Fo-Fc and Fo-Fc electron density maps of the lipid antigen α-GlcADAG contoured at the 2.2 and 0.8 σ level, respectively, in the A11B8.2 TCR-CD1d-α-GlcADAG ternary complex. (**B**) Superposition of the A11B8.2 TCR-CD1d-α-GlcADAG (firebrick), mouse Vα14-Vβ8.2 TCR-CD1d-α-GalCer (light blue), Vα10-Vβ8.1 TCR-CD1d-α-GlcCer (orange) and XV19 TCR-CD1d-sulfatide (light green) ternary complexes. For clarity, only the α1- and α2- helices of CD1d and the center of mass (spheres) of the respective TCRs are shown. (**C**) Superposition of the unligated A11B8.2 TCR (in brown) and the A11B8.2 TCR in complex with CD1d-α-GlcADAG (in light blue). For clarity, only the CDRs are shown.

Supplementary Table 1 Paired TCR α- and β-chain sequences of NKT cells identified by CD1d-α-GlcADAG tetramers. CD1d-α-GlcADAG tetramers alone or together with CD1d-α-GalCer tetramer (DP) were used to individually sort thymocytes from wt and $J\alpha18^{-/-}$ BALB/c mice. Old and IMGT TCR gene nomenclatures (nomenc.) are shown. Amino acid sequences for the CDR loops are shown. Residues partially or fully encoded by non-germline encoded nucleotides are coloured in red. The number of observations (# obs) column refers to the frequency that each unique clonotype was observed within each experiment (#1 #2 or #3).

Clone	Old nomenc. Wison, RK et al Arden, B et al	IMGT nomenclature	CDR1α	CDR2α	CDR3α	Old nomenc. Wison, RK et al Arden, B et al	IMGT nomenclature	CDR1β	CDR2β	CDR3β	# obs	Source Gate	Mouse strain
A10B8.2	Vα10 Jα50	TRAV13-3 TRAJ50	TTLNS	SPSWA	CAIASSSFSKLVF	Vβ8.2 Jβ2.1	TRBV13-2 - TRBD1 - TRBJ2-1	NNHNN	SYGAGS	CASGDWGVNYAEQFF	5 of 16	α-GlcADAG+	wt
	Vα10 Jα50	TRAV13-3 TRAJ50	TTLNS	SPSWA	CAIASSSFSKLVF	Vβ8.2 Jβ2.7	TRBV13-2 - TRBD1 - TRBJ2-7	NNHNN	SYGAGS	CASGPLWGHFEQYF	1 of 16	α-GlcADAG+	wt
	Vα15 Jα50	TRAV8-1 TRAJ50	TSITA	IRSNER	CAIASSSFSKLVF	Vβ8.2 Jβ2.5	TRBV13-2 - TRBD1 - TRBJ2-5	NNHNN	SYGAGS	CASGAAGGQDTQYF	1 of 16	α-GlcADAG+	wt
	Vα10 Jα50	TRAV13-3 TRAJ50	TTLNS	SPSWA	CAMRPSSFSKLVF	Vβ8.3 Jβ2.2	TRBV13-1 - TRBD2 - TRBJ2-2	NSHNY	SYGAGN	CASSDMGGAYTGQLYF	3 of 16	α-GlcADAG+	wt
	Vα10 Jα50	TRAV13-3 TRAJ50	TTLNS	SPSWA	CAMRPSSFSKLVFC	Vβ8.3 Jβ2.2	TRBV13-1 - TRBD2 - TRBJ2-2	NSHNY	SYGAGN	CASSDMGGAYTGQLYF	1 of 16	α-GlcADAG+	wt
	Vα10 Jα50	TRAV13-3 TRAJ50	TTLNS	SPSWA	CAMKPSSFSKLVF	Vβ8.2 Jβ2.1	TRBV13-2 - TRBD2 - TRBJ2-1	NNHNN	SYGAGS	CASGDAWDNYAEQFF	1 of 16	α-GlcADAG+	wt
	Vα14 Jα18	TRAV11 - TRAJ18	VTPDNH	LVHENDK	CVVGDRGSALGRLHF	Vβ8.2 Jβ2.7	TRBV13-2 - TRBD1 - TRBJ2-7	NNHNN	SYGAGS	CASGEWGSYEQYF	1 of 16	α-GlcADAG+	wt
	Vα4 Jα17	TRAV6-6 TRAJ17	ATSIAYPN	VITAAGQ	CALGDWTNSAGNKLTF	Vβ8.2 Jβ2.7	TRBV13-2 - TRBD1 - TRBJ2-7	NNHNN	SYGAGS	CASGDAGTGGYEQYF	1 of 6 #2	α-GlcADAG+	wt
	Vα8 Jα49	TRAV12-3 TRAJ49	TIYSNPF	SSTDNK	CALPGYQNFYF	Vβ8.2 Jβ2.2	TRBV13-2 - TRBD2 - TRBJ2-2	NNHNN	SYGAGS	CASGDLRTGQLYF	1 of 6 #2	α-GlcADAG+	wt
	Vα10 Jα50	TRAV13-3 TRAJ50	TTLNS	SPSWA	CAMRPSSFSKLVF	Vβ8.1 Jβ2.7	TRBV13-3 - TRBD1 - TRBJ2-7	NNHDY	SYYADS	CASSEPLSYEQYF	1 of 6 #2	α-GlcADAG+	wt
	Vα4 Jα50	TRAV6-5 TRAJ50	TKQYPT	VPKANE	CALSATSSSFSKLVF	Vβ8.3 Jβ2.8	TRBV13-1 - TRBD1 - TRBJ2-8	NSHNY	SYGAGN	CASSASRQGYEQYF	1 of 6 #2	α-GlcADAG+	wt
	Vα10 Jα50	TRAV13-3 TRAJ50	TTLNS	SPSWA	CAMRASSFSKLVF	Vβ8.2 Jβ2.7	TRBV13-2 - TRBD1 - TRBJ2-7	NNHNN	SYGAGS	CASGDGGEQYF	1 of 6 #2	α-GlcADAG+	wt
	Vα11 Jα50	TRAV4-3 TRAJ50	IATTT	LVPG	CAASSSSFSKLVF	Vβ8.3 Jβ2.8	TRBV13-1 - TRBD1 - TRBJ1-5	NSHNY	SYGAGN	CASNRGPNQAPLF	1 of 6 #2	α-GlcADAG+	wt
	Vα4.4 Jα50	TRAV6-6 TRAJ18	TTSIAYPN	VITAGQ	CALSDRDRGSALGRLHF	Vβ8.2 Jβ2.7	TRBV13-2 - TRBD1 - TRBJ2-7	NNHNN	SYGAGS	CASGDASSYEQYF	1 of 3 #3	α-GlcADAG+	wt
A10B8.3	Vα10 Jα50	TRAV13-3 TRAJ50	TTLNS	SPSWA	CAIASSSFSKLVF	Vβ8.3 Jβ2.1	TRBV13-1 – TRBD1 - TRBJ2-1	NSHNY	SYGAGN	CASRTGLAAEQFF	1 of 3 #3	α-GlcADAG+	wt
	Vα14 Jα18	TRAV11 - TRAJ18	VTPDNH	LVHENDK	CVVGDRGSALGRLHF	Vβ8.2 Jβ2.7	TRBV13-2 - TRBD1 - TRBJ2-7	NNHNN	SYGAGS	CASGDQGYEQYF	1 of 3 #3	α-GlcADAG+	wt
	Vα14 Jα18	TRAV11 - TRAJ18	VTPDNH	LVHENDK	CVVGDRGSALGRLHF	Vβ8.2 Jβ2.7	TRBV13-2 - TRBD2 - TRBJ2-7	NNHNN	SYGAGS	CASGDAGYEQYF	1 of 15	DP	wt
	Vα14 Jα18	TRAV11 - TRAJ18	VTPDNH	LVHENDK	CVVGDRGSALGRLHF	Vβ8.2 Jβ2.7	TRBV13-2 - TRBD2 - TRBJ2-7	NNHNN	SYGAGS	CASGDAGYEQYF	1 of 15	DP	wt
	Vα14 Jα18	TRAV11 - TRAJ18	VTPDNH	LVHENDK	CVVGDRGSALGRLHF	Vβ8.2 Jβ2.1	TRBV13-2 - TRBD1 - TRBJ2-1	NNHNN	SYGAGS	CASGEQGAAEQFF	2 of 15	DP	wt
	Vα14 Jα18	TRAV11 - TRAJ18	VTPDNH	LVHENDK	CVVGDRGSALGRLHF	Vβ7 Jβ1.5	TRBV29 - TRBD1 - TRBJ1-5	MSHET	SYDVDS	CASTPRDNQAPLF	6 of 15	DP	wt
	Vα14 Jα18	TRAV11 - TRAJ18	VTPDNH	LVHENDK	CVVGDRGSALGRLHF	Vβ8.1 Jβ2.2	TRBV13-3 - TRBD2 - TRBJ2-2	NNHDY	SYYADS	CASSDYTGQLYF	1 of 15	DP	wt
	Vα14 Jα18	TRAV11 - TRAJ18	VTPDNH	LVHENDK	CVVGDRGSALGRLHF	Vβ8.2 Jβ2.3	TRBV13-2 - TRBD1 - TRBJ2-3	NNHNN	SYGAGS	CASGDGTGYAEQFF	1 of 15	DP	wt
	Vα14 Jα18	TRAV11 - TRAJ18	VTPDNH	LVHENDK	CVVGDRGSALGRLHF	Vβ8.2 Jβ2.7	TRBV13-2 - TRBD1 - TRBJ2-7	NNHNN	SYGAGS	CASGDAGEQYF	1 of 15	DP	wt
	Vα14 Jα18	TRAV11 - TRAJ18	VTPDNH	LVHENDK	CVVGDRGSALGRLHF	Vβ8.2 Jβ2.7	TRBV13-2 - TRBD1 - TRBJ2-7	NNHNN	SYGAGS	CASGDQTVEQYF	1 of 15	DP	wt
	Vα14 Jα18	TRAV11 - TRAJ18	VTPDNH	LVHENDK	CVVGDRGSALGRLHF	Vβ8.2 Jβ2.7	TRBV13-2 - TRBD1 - TRBJ2-7	NNHNN	SYGAGS	CASGGQGSDYTF	1 of 15	DP	wt
A11B8.2	Vα11 Jα9	TRAV4-2 TRAJ9	TTMKS	LAQG	CAAVNMGYKLTF	Vβ8.2 Jβ2.7	TRBV13-2 - TRBD1 - TRBJ2-7	NNHNN	SYGAGS	CASGDPQGVSYEQYF	12 of 16	α-GlcADAG+	$J\alpha18^{-/-}$
	Vα10 Jα50	TRAV13-3 TRAJ50	TTLNS	SPSWA	CAMKSSSFSKMVF	Vβ8.2 Jβ2.7	TRBV13-2 - TRBD1 - TRBJ2-7	NNHNN	SYGAGS	CASGDAWGPYEQYF	1 of 16	α-GlcADAG+	$J\alpha18^{-/-}$
	Vα10 Jα50	TRAV13-3 TRAJ50	TTLNS	SPSWA	CAIASSSFSKLVF	Vβ8.2 Jβ2.1	TRBV13-2 - TRBD2 - TRBJ2-1	NNHNN	SYGAGS	CASGDWEYYAEQFF	1 of 16	α-GlcADAG+	$J\alpha18^{-/-}$
	Vα10 Jα50	TRAV13-3 TRAJ50	TTLNS	SPSWA	CAMRSSSFSKLVF	Vβ8.2 Jβ2.7	TRBV13-2 - TRBD1 - TRBJ2-7	NNHNN	SYGAGS	CASGDLGDYEQYF	1 of 16	α-GlcADAG+	$J\alpha18^{-/-}$
	Vα10 Jα50	TRAV13-3 TRAJ50	TTLNS	SPSWA	CAIASSSFSKLVF	Vβ8.2 Jβ1.1	TRBV13-1 – TRBD1 - TRBJ1-1	NSHNY	SYGAGN	CASRAAGNTEVFF	1 of 5 #2	α-GlcADAG+	$J\alpha18^{-/-}$
	Vα10 Jα12	TRAV13-3 TRAJ12	TTLNS	SPSWA	CAIRTGGYKVVF	Vβ8.2 Jβ2.7	TRBV13-2 – TRBD1 - TRBJ2-7	NNHNN	SYGAGS	CASGDLGTDYEQYF	1 of 5 #2	α-GlcADAG+	$J\alpha18^{-/-}$
	Vα5 Jα12	TRAV3-1 TRAJ12	DSATAY	VLSNVD	CAETGGYKVVF	Vβ8.2 Jβ1.6	TRBV13-2 – TRBD2- TRBJ1-6	NNHNN	SYGAGS	CASGDVGGMDSPLYF	1 of 5 #2	α-GlcADAG+	$J\alpha18^{-/-}$
	Vα3 Jα4	TAV9-TRAJ4	Undet	Undet	CAVRSGSFNTLF	Vβ8.2 Jβ2.7	TRBV13-2 – TRBD2 - TRBJ2-7	NNHNN	SYGAGS	CASGDADWGYEQYF	1 of 5 #2	α-GlcADAG+	$J\alpha18^{-/-}$
A17B8.2	Vα17 Jα2	TAV16-TRAJ2	TQDSSYF	QDSYKKE	CAMREGWAGGLSGKLTF	Vβ8.2 Jβ2.5	TRBV13-2 – TRBD2 - TRBJ2-5	NNHNN	SYGAGS	CASGLGGRQDTQYF	1 of 5 #2	α-GlcADAG+	$J\alpha18^{-/-}$
	Vα8 Jα16	TRAV12-3 TRAJ 16	TIYSNPF	SSTDNK	CALSEGTSSGQKLVF	Vβ8.3 Jβ2.2	TRBV13-1 - TRBD1 - TRBJ2-2	NSHNY	SYGAGN	CASRTAGQLYF	1 of 4	DP	$J\alpha18^{-/-}$
	Vα10 Jα50	TRAV13-3 TRAJ50	TTLNS	SPSWA	CAMRSSFSKLVF	Vβ8.2 Jβ2.7	TRBV13-2 - TRBD2 - TRBJ2-7	NNHNN	SYGAGS	CASGDLGDYEQYF	3 of 4	DP	$J\alpha18^{-/-}$

Supplementary Table 2. Data collection and refinement statistics

	A11B8.2-CD1d-α-GlcADAG	A11B8.2 TCR
Data collection		
Temperature	100K	100K
Resolution limits (Å)	73.04-3.0 (3.15-3.0)	45.60-1.70 (1.79-1.70)
Space Group	$P2_12_12_1$	$P2_12_12_1$
Cell dimensions (Å)	a=42.58, b=141.26, c=170.67 α=β=γ=90.00°	a=49.45, b=74.23, c=115.58 α=β=γ=90.00°
Total N$^{o.}$ observations	126122 (18431)	424889 (61496)
N$^{o.}$ unique observations	21533 (3053)	47656 (6830)
Multiplicity	5.9 (6.0)	8.9 (9.0)
Data completeness	100 (100)	100 (99.8)
Wilson B-factors (Å^2)	62.5	24.4
I/σ_I	5.3 (2.3)	16.2 (2.6)
R_{merge}	0.27 (0.80)	0.08 (0.88)
$R_{p.i.m}$ [1]	0.12 (0.32)	0.03 (0.31)
Refinement statistics		
R_{factor} [2] (%)	18.5	18.1
R_{free} [3] (%)	24.4	21.9
Non-hydrogen atoms		
- Protein	6168	3286
- Water	24	469
- Heterogen	136	-
Ramachandran plot (%)		
- Most favoured	96.39	98.54
- Allowed	3.35	1.46
B-factors (Å^2)		
- Average main chain	38	24
- Average side chain		
- α-GlcADAG	44	29
- Waters		
	53	-
	22.7	37.5
rmsd bonds (Å)	0.009	0.010
rmsd angles (°)	0.97	1.08

[1] $R_{p.i.m} = \Sigma_{hkl} [1/(N-1)]^{1/2} \Sigma_i \mid I_{hkl,\,i} - \langle I_{hkl}\rangle \mid / \Sigma_{hkl} \langle I_{hkl}\rangle$

[2] $R_{factor} = (\Sigma \mid |F_o| - |F_c| \mid) / (\Sigma |F_o|)$ - for all data except as indicated in footnote 3.

[3] 5% of data was used for the R_{free} calculation

Values in parentheses refer to the highest resolution bin.

Supplementary Table 3. A11B8.2 TCR contacts with α-GlcADAG and CD1d.

TCR gene	TCR residues	CD1d residues	Bond type
CDR1α	Thr29	Val72	VDW
CDR1α	Lys31	Asp80, Asp153	VDW
CDR1α	Lys31-Nζ	Asp80-Oδ1	SB
CDR2α	Ala51	Gln154	VDW
CDR2α	Gln52	Gln154	VDW
CDR2α	Gln52-Nε2	Gln154-Oε1	HB
CDR3α	Met93	Val75, Ser76, Arg79, Asp80	VDW
CDR3α	Gly94	Arg79, Asp80, Glu83	VDW
CDR3α	Gly94-N	Asp80-Oδ2	HB
CDR3α	Tyr95	Asp80, Glu83, Leu84, Val149, Leu150	VDW
FWα	Arg69	His68, Val72	VDW
CDR2β	Tyr50	Glu83, Lys86, Met87	VDW
CDR2β	Tyr50-Oη	Glu83-Oε1	HB
CDR3β	Pro96	Lys148, Leu145	VDW
CDR3β	Gln97	Lys148, Leu145	VDW
CDR3β	Gln97-Oε1	Lys148-Nη	HB
CDR3β	Val99	Lys148, Ala152	VDW
CDR3β	Ser100	Ala152	VDW
CDR3β	Tyr101	Val149, Ala152, Asp153	VDW
CDR3β	Tyr101-Oη	Val149-O	HB
FWβ	Tyr48	Glu83, Lys86	VDW
FWβ	Tyr48-Oη	Glu83-Oε1	HB
FWβ	Glu56	Arg21, Lys86	VDW
**TCR gene**	**TCR residues**	**α-GlcADAG atoms**	**Bond type**
CDR1α	Thr29	C1, C2, O5, O6B	VDW
CDR1α	Lys31	C10, C11	VDW
CDR1α	Lys31-Nζ	O10	HB
FWα	Lys67	C4, O3, O4	VDW

HB: Hydrogen bond, VDW: Van der Waals, SB: salt bridge. Cut-off at 4 Å for VDW interactions and 3.5 Å for HB.

Supplementary Table 4 List of Primers Used on This Study

TRAV-Primer	Sequence	Orientation
mTRAV1_Ext	GGTTATCCTGGTACCAGCA	Forward
mTRAV1_Int	CTCCACATTCCTGAGCC	Forward
mTRAV2_Ext	CATCTACTGGTACCGACAGG	Forward
mTRAV2_Int	ACTCTGAGCCTGCCCT	Forward
mTRAV3_Ext	GGCGAGCAGGTGGAG	Forward
mTRAV3_Int	GCCCTCCTCACCTGAG	Forward
mTRAV4_Ext	TCTGSTCTGAGATGCAATTTT	Forward
mTRAV4_Int	GGYTYMAGGAACAAAGGAGAAT	Forward
mTRAV5-1/5-4(D)_Ext	CTACTTCCCTTGGTATAAGCAAGA	Forward
mTRAV5-1/5-4(D)_Int	ATYCGTTCAAATATGGAAAGAAA	Forward
mTRAV6-1/6-2_Ext	CAGATGCAAGGTCAAGTGAC	Forward
mTRAV6-1/6-2_Int	GGAGAAGGTCCACAGCTC	Forward
mTRAV6-3/6-4(D)_Ext	AAGGTCCACAGCTCCTTC	Forward
mTRAV6-3/6-4(D)_Int	CAACTGCCAACAACAAGG	Forward
mTRAV6-5/6-7(D)_Ext	GTTCTGGTATGTGCAGTATCC	Forward
mTRAV6-5/6-7(D)_Int	TCCTTCCACTTGCAGAAAG	Forward
mTRAV6-6_Ext	AGATTCCGTGACTCAAACAG	Forward
mTRAV6-6_Int	ACGGCTGGCCAGAAG	Forward
mTRAV7_Ext	AGAAGGTRCAGCAGAGCCCAGAATC	Forward
mTRAV7_Int	CAKGRCYTCYYTCAACTGCAC	Forward
mTRAV8_Ext	GAGCRTCCASGAGGGTG	Forward
mTRAV8_Int	AGAGCCACCCTTGACAC	Forward
mTRAV9_Ext	CCAGTGGTTCAAGGAGTG	Forward
mTRAV9_Int	GCTTYGAGGCTGAGTTCAG	Forward
mTRAV10/10a(D)_Ext	AGAGAAGGTCGAGCAACAC	Forward
mTRAV10/10a(D)_Int	CTACACTGAGTGTTCGAGAGG	Forward
mTRAV11_Ext	AAGACCCAAGTGGAGCAG	Forward
mTRAV11_Int	AACAGGACACAGGCAAAG	Forward
mTRAV12_Ext	TGACCCAGACAGAAGGC	Forward
mTRAV12_Int	GGTTCCACGCCACTC	Forward
mTRAV13_Ext	TCCTTGGTTCTGCAGG	Forward
mTRAV13_Int	TGCAGGAGGGGGAGA	Forward
mTRAV14_Ext	GCAGCAGGTGAGACAAAG	Forward
mTRAV14_Int	CTCTGACAGTCTGGGAAGG	Forward
mTRAV15_Ext	CASCTTYTTAGTGGAGAGATGG	Forward
mTRAV15_Int	AYTCTGTAGTCTTCCAGAAATCAC	Forward
mTRAV16_Ext	GTACAAGCAAACAGCAAGTG	Forward
mTRAV16_Int	ATTATTCTCTGAACTTTCAGAAGC	Forward
mTRAV17_Ext	CAGTCCGTGGACCAGC	Forward
mTRAV17_Int	TATGAAGGAGCCTCCCTG	Forward
mTRAV18_Ext	AACGGCTGGAGCAGAG	Forward
mTRAV18_Int	CAAGATTTCACCGCACG	Forward
mTRAV19_Ext	GCAAGTTAAACAAAGCTCTCC	Forward
mTRAV19_Int	GCTGACTGTTCAAGAGGGA	Forward
mTRAV21_Ext	GTGCACTTGCCTTGTAGC	Forward
mTRAV21_Int	AATAGTATGGCTTTCCTGGC	Forward
mTRAC_Ext	GGCATCACAGGGAACG	Reverse
mTRAC_Int	GCACATTGATTTGGGAGTC	Reverse

TRBV-Primer	Sequence	Orientation
mTRBV1_Ext	TACCACGTGGTCAAGCTG	Forward
mTRBV1_Int	GTATCCCTGGATGAGCTG	Forward
mTRBV2_Ext	CAGTATCTAGGCCACAATGC	Forward
mTRBV2_Int	GGACAATCAGACTGCCTC	Forward
mTRBV3_Ext	CCCAAAGTCTTACAGATCCC	Forward
mTRBV3_Int	GATATGGGGCAGATGGTG	Forward
mTRBV4_Ext	GACGGCTGTTTTCCAGAC	Forward
mTRBV4_Int	CAGGTGGGAAATGAAGTG	Forward
mTRBV5_Ext	GGTATAAACAGAGCGCTGAG	Forward
mTRBV5_Int	GCCAGAGCTCATGTTTCTC	Forward
mTRBV12_Ext	GGGGTTGTCCAGTCTCC	Forward
mTRBV12_Int	CCAGCAGATTCTCAGTCC	Forward
mTRBV13_Ext	GCTGCAGTCACCCAAAG	Forward
mTRBV13_Int	GTACTGGTATCGGCAGGAC	Forward
mTRBV14_Ext	GCAGTCCTACAGGAAGGG	Forward
mTRBV14_Int	GGTATCAGCAGCCCAGAG	Forward
mTRBV15_Ext	GAGTTACCCAGACACCCAG	Forward
mTRBV15_Int	GTGTGAGCCAGTTTCAGG	Forward
mTRBV16_Ext	CCTAGGCACAAGGTGACAG	Forward
mTRBV16_Int	GAAGCAACTCTGTGGTGTG	Forward
mTRBV17_Ext	GAAGCCAAACCAAGCAC	Forward
mTRBV17_Int	GAACAGGGAAGCTGACAC	Forward
mTRBV19_Ext	GATTGGTCAGGAAGGGC	Forward
mTRBV19_Int	GGTACCGACAGGATTCAG	Forward
mTRBV20_Ext	GGATGGAGTGTCAAGCTG	Forward
mTRBV20_Int	GCTTGGTATCGTCAATCG	Forward
mTRBV23_Ext	CTGCAGTTACACAGAAGCC	Forward
mTRBV23_Int	GCCAGGAAGCAGAGATG	Forward
mTRBV24_Ext	CAGACTCCACGATACCTGG	Forward
mTRBV24_Int	GCACACTGCCTTTTACTGG	Forward
mTRBV26_Ext	GGTGAAAGGGCAAGGAC	Forward
mTRBV26_Int	GAGGTGTATCCCTGAAAAGG	Forward
mTRBV29_Ext	GCTGGAATGTGGACAGG	Forward
mTRBV29_Int	GTACTGGTATCGACAAGACCC	Forward
mTRBV30_Ext	CCTCCTCTACCAAAAGCC	Forward
mTRBV30_Int	GGACATCTGTCAAAGTGGC	Forward
mTRBV31_Ext	CTAACCTCTACTGGTACTGGCAG	Forward
mTRBV31_Int	CTGTTGGCCAGGTAGAGTC	Forward
mTRBC_Ext	CCAGAAGGTAGCAGAGACCC	Reverse
mTRBC_Int	GGGTAGCCTTTTGTTTGTTTG	Reverse

Vector-Primer	Sequence	Orientation
pUC57_fwd	AGGCGATTAAGTTGGGTAAC	Forward
pUC57_rev	CGTATGTTGTGTGGAATTGTAG	Forward
pMSCV_fwd	TTGAACCTCCTCGTTCGACC	Reverse
pMSCV_rev	CATATAGACAAACGCACACC	Reverse
pET_Fwd	CTAGTTATTGCTCAGCGG	Forward
pET_Rev	CTAGTTATTGCTCAGCGG	Reverse
