# Supplementary material for: Distinct CD1d docking strategies exhibited by diverse Type II NKT cell receptors
Source: Nat Commun. 2019 Nov 20;10:5242. doi: 10.1038/s41467-019-12941-9 (PMC6868179; doi:10.1038/s41467-019-12941-9)
Supplement: Supplementary file 3 — Reporting Summary [file 41467_2019_12941_MOESM3_ESM.pdf]

nature research

Corresponding author(s): Jamie Rossjohn

Last updated by author(s): Oct 10, 2019

Reporting Summary

Nature Research wishes to improve the reproducibility of the work that we publish. This form provides structure for consistency and transparency in reporting. For further information on Nature Research policies, see Authors & Referees and the Editorial Policy Checklist.

Statistics

For all statistical analyses, confirm that the following items are present in the figure legend, table legend, main text, or Methods section.

n/a	Confirmed	
☐	☒	The exact sample size (n) for each experimental group/condition, given as a discrete number and unit of measurement
☐	☒	A statement on whether measurements were taken from distinct samples or whether the same sample was measured repeatedly
☐	☒	The statistical test(s) used AND whether they are one- or two-sided<br>*Only common tests should be described solely by name; describe more complex techniques in the Methods section.*
☒	☐	A description of all covariates tested
☒	☐	A description of any assumptions or corrections, such as tests of normality and adjustment for multiple comparisons
☐	☒	A full description of the statistical parameters including central tendency (e.g. means) or other basic estimates (e.g. regression coefficient) AND variation (e.g. standard deviation) or associated estimates of uncertainty (e.g. confidence intervals)
☒	☐	For null hypothesis testing, the test statistic (e.g. F, t, r) with confidence intervals, effect sizes, degrees of freedom and P value noted<br>*Give P values as exact values whenever suitable.*
☒	☐	For Bayesian analysis, information on the choice of priors and Markov chain Monte Carlo settings
☒	☐	For hierarchical and complex designs, identification of the appropriate level for tests and full reporting of outcomes
☒	☐	Estimates of effect sizes (e.g. Cohen's d, Pearson's r), indicating how they were calculated

Our web collection on statistics for biologists contains articles on many of the points above.

Software and code

Policy information about availability of computer code

Data collection	n/a
Data analysis	n/a

For manuscripts utilizing custom algorithms or software that are central to the research but not yet described in published literature, software must be made available to editors/reviewers. We strongly encourage code deposition in a community repository (e.g. GitHub). See the Nature Research guidelines for submitting code & software for further information.

Data

Policy information about availability of data

All manuscripts must include a data availability statement. This statement should provide the following information, where applicable:

- Accession codes, unique identifiers, or web links for publicly available datasets
- A list of figures that have associated raw data
- A description of any restrictions on data availability

Accession codes
The A11B8.2 NKT TCR and A11B8.2 TCR-CD1d-GlcADAG ternary complex were deposited in the Protein Data Bank (PDB) under the accession codes 6MRA and 6MSS, respectively.

Field-specific reporting

Please select the one below that is the best fit for your research. If you are not sure, read the appropriate sections before making your selection.

☒ Life sciences ☐ Behavioural & social sciences ☐ Ecological, evolutionary & environmental sciences

For a reference copy of the document with all sections, see nature.com/documents/nr-reporting-summary-flat.pdf

Life sciences study design

All studies must disclose on these points even when the disclosure is negative.

Sample size	Described in all figure legends
Data exclusions	n/a
Replication	All experiments were reproduced at least twice, in some cases dose responses were further use for selecting optimal dose o stimulation for activation assays.
Randomization	No Randomization was used
Blinding	No Binding done

Reporting for specific materials, systems and methods

We require information from authors about some types of materials, experimental systems and methods used in many studies. Here, indicate whether each material, system or method listed is relevant to your study. If you are not sure if a list item applies to your research, read the appropriate section before selecting a response.

Materials & experimental systems

n/a	Involved in the study	
☐	☒	Antibodies
☐	☒	Eukaryotic cell lines
☒	☐	Palaeontology
☐	☒	Animals and other organisms
☒	☐	Human research participants
☒	☐	Clinical data

Methods

n/a	Involved in the study	
☒	☐	ChIP-seq
☐	☒	Flow cytometry
☒	☐	MRI-based neuroimaging

Antibodies

Antibodies used	Listed in methods section
Validation	All antibodies used in this study are commercially available and have been validated for use for flow cytometry as per vendor websites. All clones and companies are provided in the materials and methods section. Each antibody is titrated in house on appropriate cell types prior to use in experiments.

Eukaryotic cell lines

Policy information about cell lines

Cell line source(s)	HEK293T and BW58 cells have been in stocked in the laboratory for approx. 20 years. Original source is unknown.
Authentication	293T cells have not been validated , though they morphologically and functionally resemble 293T cells. BW58 cell lines have been validated by our collaborators.
Mycoplasma contamination	All cell lines used tested negative for mycoplasma
Commonly misidentified lines (See ICLAC register)	n/A

Animals and other organisms

Policy information about studies involving animals; ARRIVE guidelines recommended for reporting animal research

Laboratory animals	BALB/c wt, BALB/c Jα18-/-, BALB/c CD1d-/-, female 8-14 weeks,
Wild animals	No wild animals
Field-collected samples	n/a
Ethics oversight	Animals were bred and maintained according to ethics approval from The University of Melbourne Animal Ethics Committee, under specific pathogen-free conditions at the Biological Research Facility at the Department of Microbiology and Immunology, or by the Animal Resources Centre (ARC; Canning Vale, WA, Australia).

Note that full information on the approval of the study protocol must also be provided in the manuscript.

Flow Cytometry

Plots

Confirm that:

- [x] The axis labels state the marker and fluorochrome used (e.g. CD4-FITC).
- [x] The axis scales are clearly visible. Include numbers along axes only for bottom left plot of group (a 'group' is an analysis of identical markers).
- [ ] All plots are contour plots with outliers or pseudocolor plots.
- [x] A numerical value for number of cells or percentage (with statistics) is provided.

Methodology

Sample preparation	As detailed in methods section
Instrument	As detailed in methods section for flow cytometry, SPR, or crystallography, soluble TCR generation.
Software	As detailed in methods section
Cell population abundance	As detailed in each figure legend.
Gating strategy	As detailed in each figure legend

- [x] Tick this box to confirm that a figure exemplifying the gating strategy is provided in the Supplementary Information.
